# Performance Evaluation of Mixed Reality Display for Guidance During Transcatheter Cardiac Mapping and Ablation

**DOI:** 10.1109/JTEHM.2020.3007031

**Published:** 2020-07-03

**Authors:** Michael K. Southworth, Jennifer N. Avari Silva, Walter M. Blume, George F. Van Hare, Aarti S. Dalal, Jonathan R. Silva

**Affiliations:** 1 SentiAR, Inc.St. LouisMO63108USA; 2 Department of PediatricsSchool of MedicineWashington University in St. Louis 7284 St. LouisMO63130USA; 3 Department of Biomedical EngineeringWashington University in St. Louis 7284 St. LouisMO63130USA

**Keywords:** Augmented reality, cardiology, head-mounted displays, minimally invasive surgery, mixed reality

## Abstract

Cardiac electrophysiology procedures present the physician with a wealth of 3D information, typically presented on fixed 2D monitors. New developments in wearable mixed reality displays offer the potential to simplify and enhance 3D visualization while providing hands-free, dynamic control of devices within the procedure room. Objective: This work aims to evaluate the performance and quality of a mixed reality system designed for intraprocedural use in cardiac electrophysiology. Method: The Enhanced Electrophysiology Visualization and Interaction System (ĒLVIS) mixed reality system performance criteria, including image quality, hardware performance, and usability were evaluated using existing display validation procedures adapted to the electrophysiology specific use case. Additional performance and user validation were performed through a 10 patient, in-human observational study, the Engineering ĒLVIS (E2) Study. Results: The ĒLVIS system achieved acceptable frame rate, latency, and battery runtime with acceptable dynamic range and depth distortion as well as minimal geometric distortion. Bench testing results corresponded with physician feedback in the observational study, and potential improvements in geometric understanding were noted. Conclusion: The ĒLVIS system, based on current commercially available mixed reality hardware, is capable of meeting the hardware performance, image quality, and usability requirements of the electroanatomic mapping display for intraprocedural, real-time use in electrophysiology procedures. Verifying off the shelf mixed reality hardware for specific clinical use can accelerate the adoption of this transformative technology and provide novel visualization, understanding, and control of clinically relevant data in real-time.

## Graphical abstract

This work evaluates the performance and quality of a mixed reality system designed for intraprocedural use in cardiac electrophysiology. We tested the Enhanced Electrophysiology Visualization and Interaction System (ĒLVIS)'s image quality, hardware performance, and usability in 10 patients. The ĒLVIS system achieved acceptable frame rate, latency, and battery runtime with acceptable dynamic range and depth distortion as well as minimal geometric distortion. Bench testing results corresponded with physician feedback in the observational study. The ĒLVIS system, based on current commercially available mixed reality hardware, is capable of meeting the hardware performance, image quality, and usability requirements of the electroanatomic mapping display for intraprocedural, real-time use in electrophysiology procedures.

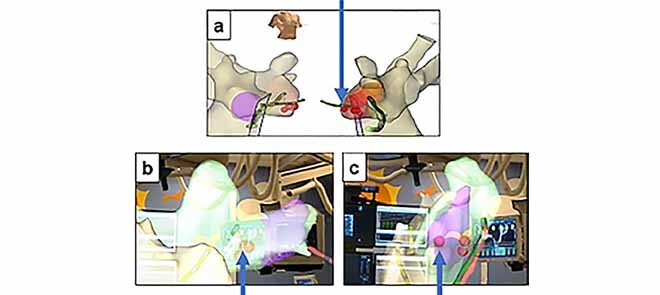

## I. Introduction

Minimally invasive surgical procedures have vastly improved patient outcomes by minimizing trauma, miniaturizing wounds, accelerating recovery and reducing hospital stays. However, the lack of an open surgical site means that there is no direct visual access for the surgeon to interact with the target anatomy. In response, sophisticated navigation systems have been developed to sense and display the position of the surgeon’s tools relative to the patient’s anatomy [1]–[5] and have gained wide adoption. Despite these technical advances, interventional physicians who are performing minimally invasive procedures experience significantly greater mental fatigue in comparison to those who perform open procedures [6]–[8]. The mental burden of processing these additional data and reconstructing the 3-dimensional (3D) features of the patient anatomy from orthogonal 2-dimensional (2D) displays is a significant contributor to this fatigue.

As new sources of data are added to support new capabilities within the procedure, the technological complexity of these procedures grows. This generally requires a commensurate expansion of the supporting team to help compensate for the additional processing and interaction loads of the various systems. Physicians typically rely on oral commands to these supporting technicians to manipulate the data presented in these navigation systems in order to maintain control of the instruments and maintain sterility. The resulting lack of direct physician control and imprecision in verbal commands [9] can lead to tense situations and increase user frustration; or worse, result in medical error [10]. Additionally, systems are decentralized and isolated, with each component requiring a separate monitor to display data, regardless of whether it is currently being utilized. This abundance of information can lead to physician overstimulation through data overload [11], [12].

New developments in an augmented reality technology called mixed reality, where virtual displays are integrated with the physical world [13]–[15], have demonstrated the potential to address these challenges in visualization and provide sterile, dynamic control of complex 3D data through gaze, gesture and voice interactions for medical applications [14]. Fixed 2D viewing monitors are replaced with 3D information presented on dynamic 3D stereoscopic displays that can be positioned responsive to changing user preferences. Although mixed reality advances the ability to visualize and control information, widespread adoption in open surgical procedures, specifically regarding surgical overlay, still faces several practical challenges [16]. However, minimally invasive procedures can benefit from the direct visualization and control of complex information in mixed reality without requiring the overlay of virtual data on physical anatomy [17]–[19].

Cardiac electrophysiology (EP) procedures are highly complex and require continuous assessment of multiple sources of information. Over the course of a procedure, electrophysiologists place multiple catheters within the heart, using electrical signals to induce, diagnose and ablate abnormal electrical foci that cause cardiac arrhythmias. These procedures have been enhanced significantly by the development of electroanatomic mapping systems (EAMS) that provide a real-time display of all catheter locations within a cardiac geometry. EAMSs also construct a 3D map of the interior surface of the heart (endocardium) incorporating both anatomic location and local electrical signal. As in most minimally invasive procedures, the 3D information in maps is limited to display on conventional 2D screens that require the clinician to reconstruct, interpret, and maintain a mental model of the 3D anatomy throughout the procedure.

**FIGURE 1.**
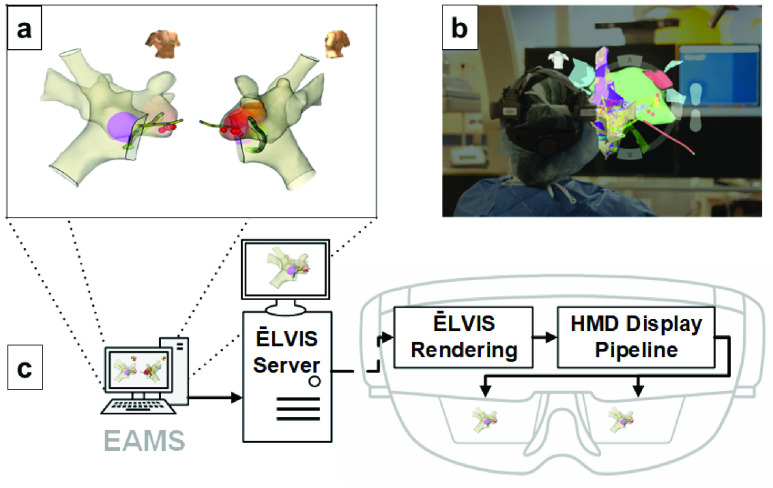
ĒLVIS 3D mixed reality pipeline. The ĒLVIS mixed reality pipeline converts generated 3D map information that is shown as 2D projections on the electroanatomic mapping system (EAMS) display (a) into 3D stereoscopic representations that appear suspended within the EP laboratory environment (b) Operators may walk or peer around the 3D model of real-time diagnostic mapping information, as if it were anchored in the real world (Simulated image). (c) 3D geometry, annotation and tool position data is exported from the EAMS to the ĒLVIS server for format translation, encryption and compression, and is transmitted wirelessly to the Head mounted display (HMD). The HMD software renders the 3D data to the HMD stereoscopic display pipeline.

The Enhanced Electrophysiology Visualization and Interaction System (ĒLVIS) system was developed to provide electrophysiologists with virtual 3D data placed within the clinical work area in stereoscopic 3D display (**Fig. 1**). The system employs an optical see through (OST), near eye display (NED), head mounted device (HMD, Microsoft HoloLens) with custom HMD rendering software to provide a mixed reality system for the physician. The HMD can be worn with or without prescription lenses (including leaded lenses) and is not tethered or cabled. The headset uses multiple sensors to continuously measure its position within the room such that displayed 3D images remain virtually anchored in the physical space. The HMD is integrated into the EP suite through an off the shelf wireless access point and medical computer and receives real-time, exported 3D data from the EAMS.

In order to use the HMD to support medical procedures, the image quality characteristics of the system must be quantified. Although the evaluation of these new display systems is an area of active research within regulatory bodies [20] and standards organizations [21], standard evaluations do not yet exist, and may not be appropriate for every use case. To evaluate the ĒLVIS system, a set of tests were developed to evaluate the intrinsic characteristics of the display hardware, rendering pipeline and user perception of the presented 3D data based on guidelines for traditional 2D displays [22]. To support the acceptability of the display modality for displaying color keyed 3D anatomic data, human perception of these stereoscopic 3D images (“human-in-the-loop” testing) was assessed. In addition to these bench tests, a first-in-man [23] observational study to validated and assess the ability of this system to be safely and beneficially used for cardiac EP procedures was performed.

## II. Materials and Methods

### A. Image Quality

Display accuracy characteristics were evaluated to determine the suitability of the HMD for clinical use. Due to the novel nature of the OST and stereoscopic 3D NED of the HMD, testing of the display was focused on qualitative and quantitative measurement of a subset of display parameters unique to this type of display. The display generates a 3D image by displaying a stereoscopic pair of images, one to each eye, to simulate depth. The tests first evaluated geometric distortion of a single display with synthetic test patterns to quantify geometric distortion of the display chain, and representative anatomy to evaluate the anatomical rendering pipeline. After single display evaluation, the perception of the stereoscopic pair of displays was evaluated by including the 3D interpretation of an operator in “human in the loop” testing. Additional qualitative human in the loop testing evaluated the color representation of the display in relevant lighting conditions to characterize the dynamic range of the display. The following tests were designed to evaluate these aspects of the display.

#### 1) Geometric Distortion

**FIGURE 2.**
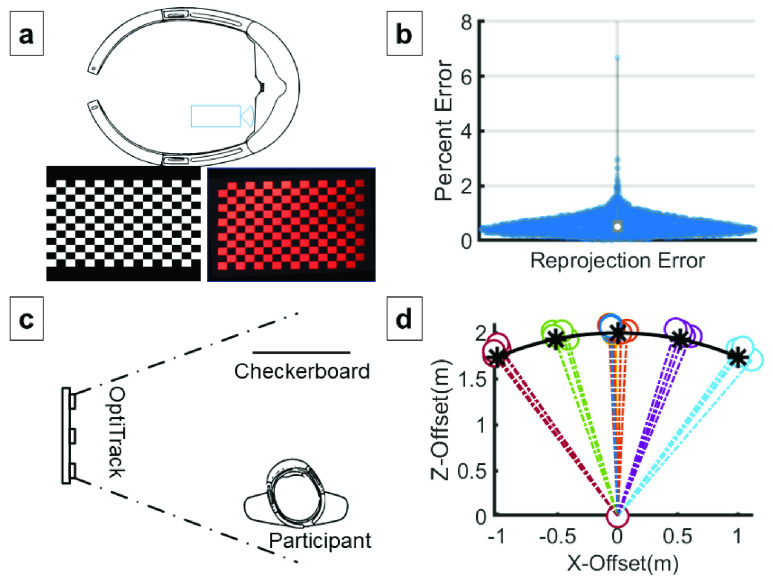
Image Quality (a) Head mounted display (HMD) geometric distortion error was measured by displaying a $16\times 12$ checkerboard image (red channel only) of known dimensions and capturing its resulting display with a calibrated camera placed at user’s eye position. Twelve different checkerboard poses were captured. The captured images were processed by a corner detection algorithm to generate detected positions for comparison against the known corner positions in the reference images. The corner positions were least-square fit by a geometric transform for all patterns compensate for camera misalignment to produce the error distribution shown, normalized to the checkerboard size (b). Mean, SD of reprojection error was 0.52%±0.31%. An erroneous corner detection result in one of the images caused a single false 6.64% distortion outlier. (c) During human in the loop accuracy testing, a user aligned virtual checkerboard images placed at various orientations with a physical checkerboard poster affixed to a wall at eye level. An optical tracker with clear line of sight to user and target, recorded the position of the user as they moved to align the image. (d) The summary plot shows the results for seven different image poses of the virtual checkerboard. Each color represents a single image pose position recorded across two calibrated HMDs and two subjects. The positions represent the relative position of subject relative to the physical image at (0,0) in order to superimpose the virtual checkerboard image. The arc shows the specified distance that the virtual object was rendered at within the HMD, and * symbols show expected positions. Note the bias of measured positions away from the origin.

The HMD renders images on small liquid crystal on silicon (LCoS) displays, which are then propagated through image forming optics, in-coupled into waveguides, expanded in 2 dimensions, and out-coupled through diffraction gratings to form a stereoscopic image to each eye [24]. Compensation for distortion is performed in software by the HMD graphics processor [24]. The residual geometric distortion of this optical display pipeline was evaluated using procedures adapted from AAPM TG18 [22] for the evaluation of cathode ray tube displays. The HMD display output of a $16\times 12$ checkerboard was captured using a calibrated camera capturing images through the display of the HMD for 12 rotations about the vertical, horizontal, and forward axes. These images were assessed for geometric distortion for each checkerboard square and globally across the entire test pattern (**Fig. 2a**).

#### 2) Application Specific Rendering Distortion

**FIGURE 3.**
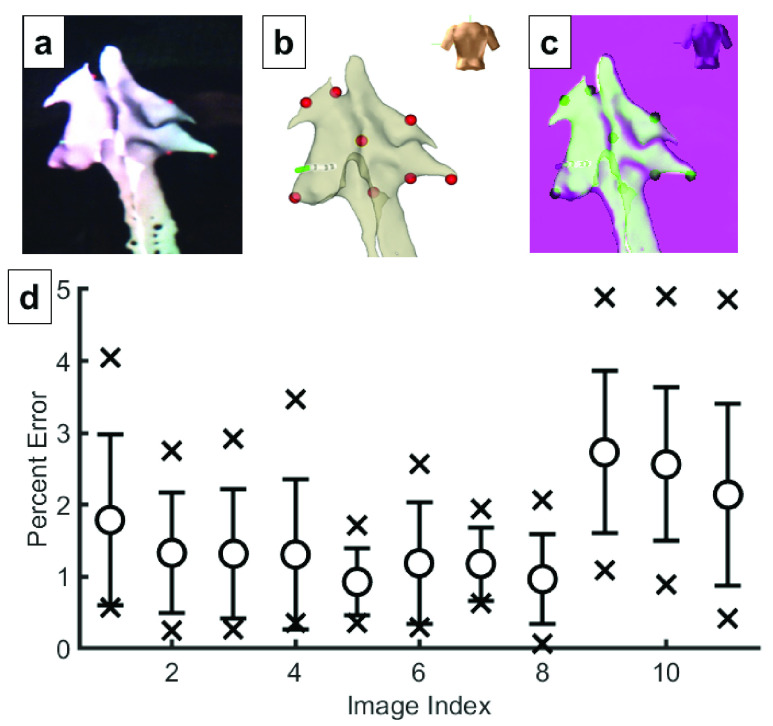
Application Specific Rendering Distortion. To assess similarity to an electroanatomic mapping system (EAMS display, we compared through the lens capture from the head mounted display (HMD) display (a) with screenshots exported from the EAMS system (b). The two images were then registered for comparison (c). Red markers show positions of landmarks that were annotated on the geometry via the EAMS to facilitate registration. A total of 11 image pairs employing anterior-posterior, posterior-anterior, left-anterior-oblique, and right-anterior oblique viewing directions were evaluated, corresponding to standard electrophysiological views. The computed reprojection error for the registered landmarks from HMD into EAMS is shown in (d). Extrema, mean and standard deviation are presented for all 11 registrations. Overall mean and SD of error was 1.6%±1.1%.

EAMSs display 3D mapping information on 2D monitors for electrophysiologists to diagnose arrhythmias and to guide placement of cardiac ablations for treatment. Geometric distortion testing evaluated the HMD with regular, planar test data. To further evaluate the consistency of the HMD rendering pipeline and display architecture with respect to the EAMS 2D display, captured images of the HMD display were compared with the corresponding EAMS display output. Similarity was evaluated by using the EAMS to place markers at anatomic landmarks visible in both systems and registering the standard 2D EAMS mapping view directions with their 3D HMD display equivalents (**Fig. 3**). The reprojection error of these landmarks was then compared to evaluate any application-specific geometric distortion error that may be introduced in the anatomic rendering pipeline and not encompassed in the previous geometric distortion tests.

#### 3) Human-in-the-Loop Virtual Object Positioning Accuracy

The stereoscopic rendering of the HMD produces the 3D representation of an object placed in space and is affected by the 3D tracking of the HMD, the calibrated interpupillary distance (IPD), and any intrinsic errors within the display. Although the EP use case does not require superimposition of the virtual image onto physical objects or anatomy, this is particularly important for other potential applications that require registration of imagery or image overlays, where position, vergence, accommodation, and scale must match closely. The accuracy with which the HMD can position a virtual image combined with the perception of depth with a human in the loop was characterized by tracking the physical position of users as they attempted to superimpose a virtual image on a physical image of the same dimensions for 7 different checkerboard poses. Two error measures were assessed: 1) the error in angulation, and 2) the error in distance from the perceived object, termed “scale”.

#### 4) Dynamic Range Evaluation

**FIGURE 4.**
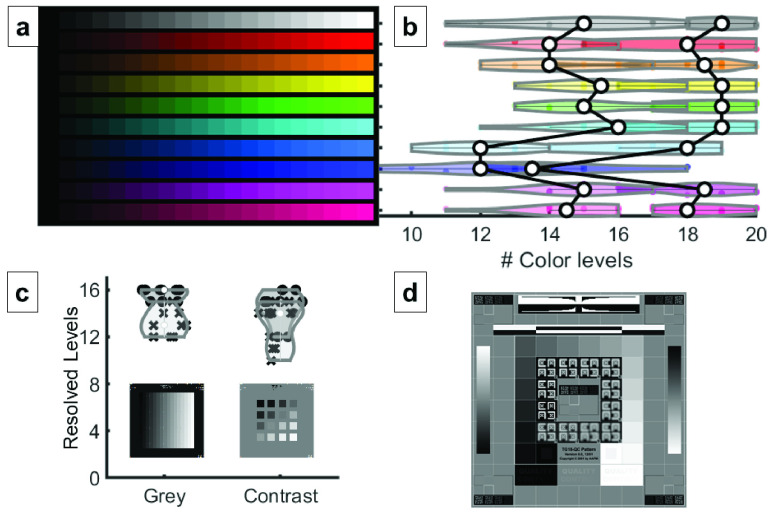
Dynamic Range Evaluation. Test patterns were shown to 10 observers at a fixed rendering distance, perpendicular to the user, and filled the usable field of view of the HMD. (a) Colorbar test chart used in the evaluation, showing ten different color hues, each with 20 levels of brightness. (b) Results show the mean and distribution of observable levels across observers for each color hue for bright (left plot) and darkened (right plot) room conditions. A larger number of levels were distinguishable in darkened room conditions. (c) Grey level and contrast evaluation images were shown to observers. The input images are shown with the distribution of observable levels in brightly lit rooms (x’s) and darkened rooms (circles) plotted above them. (d) Subjects were also asked to qualitatively evaluate their perception of the AAPM-TG18-QC pattern [27] in light and dark conditions. Darkened room conditions produced better viewing results.

In an EP procedure, as a catheter with one or more electrodes is moved throughout the heart anatomy, the EAMS records the 3D position, signal voltage magnitude and timing relative to a reference signal of the electrodes to build a 3D geometric representation with corresponding electrical data. The voltage or timing data can then be encoded as a color shown at that position of the map and interpolated between recorded positions to produce a 3D map of electrical information. The EP lab uses different lighting conditions depending on the task and clinician preference, and dimmer lighting may be used in order to better view monitors or brighter lighting may be required during surgical access to introduce catheters. The optics of virtual and augmented reality displays often introduce color artifacts that may affect the presentation of color [25], and optical see through display contrast is affected by ambient lighting conditions [26]. Although high color accuracy is not required for the interpretation of EAMS models, evaluating the performance of the HMD will aid appropriate human factors guidelines. To understand and quantify the ability of HMD to convey color and greyscale information within the dynamic lighting conditions of the cardiac catheterization lab, perceived dynamic range of color and grayscale test patterns was evaluated user under both darkened and ambient room conditions (**Fig. 4**).

### B. Hardware Performance

Latency, display frame rates and battery runtime have previously presented significant barriers to widespread adoption of virtual and mixed reality displays across most real-time applications [28], [29]. As technology has advanced, specific medical applications have become potentially feasible for use an intraprocedural context. To address the specific requirements of an EP procedure, EP application specific latency, frame rate, and battery runtime metrics were evaluated.

#### 1) Latency

Latency, or delay of data transfer in the system, is an important variable to minimize for clinical adoption [30]. This is particularly true when the user is moving a catheter while monitoring the corresponding catheter navigation on a computer display. The overall latency of the EAMS to ĒLVIS display was measured by using the through the display camera capture configuration used in the Image Quality testing **(Fig. 2a)**. The high-speed camera mounted behind the HMD display captured both the ĒLVIS model through the HMD and the EAMS computer monitor simultaneously. The number of elapsed frames between the appearance of a marker on the EAMS and the appearance of the marker on the virtual model was measured for 11 markers on the HMD and EAMS. This configuration measures the latency of the EAMS in transmission to ĒLVIS, and additional latency within ĒLVIS in translation, encoding, transmission, decryption, rendering, and display.

#### 2) Frame Rate

**FIGURE 5.**
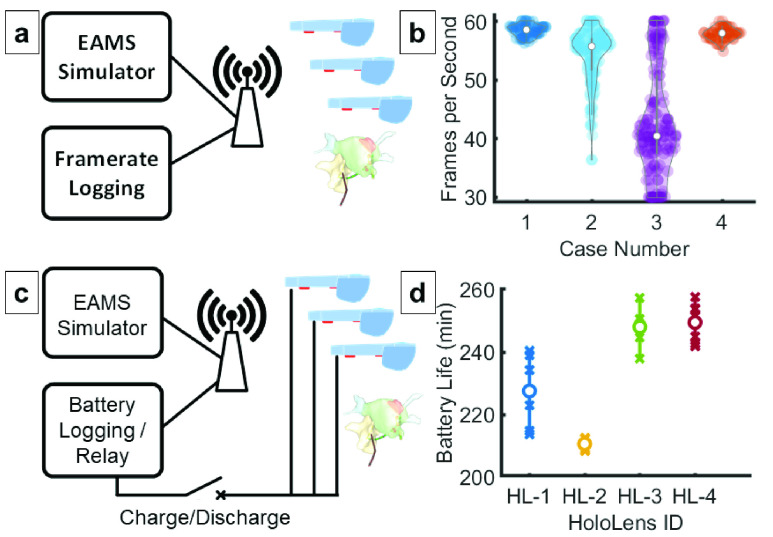
Frame Rate and Battery Life Evaluation $\vert $ The ĒLVIS rendering performance was evaluated by simulating an EAMS with pre-recorded case data. (a) The largest models from 4 recorded cases were played back at 1Hz to simulate the expected worst-case processing load and model complexity. Catheter positions were updated at the nominally expected 20Hz. (b) Resulting frame rate distributions are shown for each case with median values represented by white circles. The average frame rate across all simulations was 53.5± 5.4 frames per second. Battery life was evaluated using an EAMS simulation with logger that was connected to the normal ĒLVIS undergoing testing. (c) The HMD was then attached to either an external battery and a network-controlled relay that allowed the test fixture to cycle the devices through charge and discharge cycles. A recorded electrophysiology case was then simulated repeatedly through the EAMS simulator at either normal playback speed or at the maximum network throughput for stress testing. (d) Battery testing performed on four HMDs across 7 discharge cycles; X’s show the distribution, white circles show mean values.

Low frame rendering rates result in uneven motion, distortion of images on the 3D display, and potentially user discomfort [31]. This is especially noticeable in sequences with motion or with large user head movements. To evaluate the frame rate performance of ĒLVIS, the 10 highest polygon EAMS models were selected from 4 clinical recordings. These models were repeatedly transmitted at 1 Hz along with catheter position information at 20 Hz to simulate a representative high load scenario. A 30 frames per second (FPS) threshold was selected based on the limited movement of the wearer during interventional cardiac procedures and design guidelines for MR applications (**See Fig. 5b**).

#### 3) Battery Runtime

EP procedure times are variable, and depending on patient and substrate can range from one hour to over eight hours in length [32]. To evaluate the ability of the ĒLVIS system to support an EP procedure, HMDs were subjected to runtime testing using a simulated EP case while charging and discharging with and without 38 Wh and 48.1 Wh supplemental batteries. 4 HMDs were subjected to 7 full charge and discharge cycles while replaying a simulated case. Feasibility of using supplemental batteries was evaluated on a single HMD for each battery size during simulated case playback until fully discharged.

### C. Engineering ĒLVIS Study (E2 Study)

Following successful accuracy and performance testing, clinical feasibility user validation was performed in the Engineering ĒLVIS (E2) Study. After obtaining approval from the Washington University School of Medicine Institutional Review Board (IRB), patients with a clinical indication for a transcatheter EP study (EPS) were enrolled in the study protocol. Informed consent and assent were obtained as indicated. Patients underwent EPS using currently available equipment as normal according to the standard of care. During these procedures a separate second team of observers, comprised of a pediatric electrophysiologist (“Observing EP”) and an engineer, were located outside the EP laboratory in the control room, and had the ability to observe the procedure both through a window and through the HMD. There was no communication between the observing EP and the electrophysiologist who was performing the procedure (“Performing EP”). The observing team had access to the ĒLVIS and the observed geometry creation, electroanatomic map creation, catheter manipulations, and catheter positioning viewable through the HMD. The observing EP provided user feedback during the case to the engineer.

At the conclusion of the procedure, both the performing EP and the observing EP were provided access to the recorded case through the HMD and could review the case, geometries created, and placement of lesion markers. Both the observing EP and the performing EP were asked to review the geometry and maps created in the EAMS as well as the ĒLVIS for qualitative accuracy.

### D. Hardware Materials

The Microsoft HoloLens 1 HMD, Model 1688, operating on the Microsoft Windows 10.0.14393 operating system was used for all investigations. The HoloLens contains an Intel Atom $\times 5$ general purpose central processor and a custom graphics processor with DirectX support for rendering. The display consists of two liquid-crystal-on-silicon (LCoS) displays supporting $1268 \times 720$ pixels over an approximate 34 degrees of diagonal field of view. These LCoS displays are coupled into a pair of image forming optics and waveguides to scale and focus the image and expand the exit pupil for display to each eye, and accommodate individual IPDs. The HMD supports primary wireless communication over 802.11ac Wi-Fi.

To capture through the display images, a fixture consisting of a FLIR Systems Chameleon3 global shutter, color, high speed camera (CM3-U3-13Y3C-CS) with 8mm lens (Marshall V-4608-CS-IRC) to capture HMD images at up to 1.3 MP and 149 Hz. The camera assembly was rigidly affixed to the HMD using an inverted, 3D printed “heavy spectator mount” [33]. This fixture was used for capturing simulated case playback data for framerate evaluation as well as real-time case data for latency evaluation. A luminance meter (TES-137) directed at a Savage Wide Tone Super White #1 background was used to measure incident HMD lighting. Dynamic range HMD images were displayed in front of the background paper for evaluation.

Optical tracking of the HMD and physical target images was performed using an OptiTrak V120 Trio optical tracker system and 3M spherical markers affixed to the HMD and checkerboard targets.

The battery runtime evaluation fixture consisted of a software controlled alternating current relay (ControlByWeb WebSwitch XRDI-WS3) coupled with custom software to control power to the AC supply, the HMD powered state and log reported charge rate, discharge rate, and remaining capacity of the internal battery. Supplemental batteries for the evaluation of runtime extension were selected based on regulated output voltage to most closely match the original equipment HMD charger (13 W, 2.5A at 5.2V). Tests were performed with a 10,000 mAH 5V at 2.4A, 38 Wh (Ubio Labs Model: 253685) and 13000 mAH 5V at 3A 48.1 Wh (Anker Model 79AN7925) external batteries connected to the standard HMD charging port.

The EAMS used for latency testing and observational clinical studies was the Abbott/St Jude Medical EnSite Velocity EAMS (St Jude Medical, Minneapolis, MN), using the suite of normal catheters.

### E. Software Materials

Custom Universal Windows Platform (UWP) applications were developed in C++/DirectX for the evaluation of hardware performance, displaying test patterns for the evaluation of geometric distortion, and object positioning accuracy. For all other configurations, the unmodified ĒLVIS UWP application was used on the HMD for network communications and rendering. Additional Windows C# monitoring applications were developed to support the collection of system metrics including framerate, number of polygons and battery metrics during the E2 Study as well as to support controlling peripherals in the battery runtime fixture.

Images were captured in the through-the-display capture fixture using FLIR SpinView software in motion JPG format to minimize frame to frame artifacts. VirtualDub was used to decompose videos into individual frames for further analysis and for counting the number of elapsed between events for latency calculations. Lens corrections were calculated and applied to individual frames using the Matlab Computer Vision and Image Processing toolbox (R2017b). All registrations and reprojections were performed using these Matlab toolboxes.

Custom test patterns were generated using Matlab and exported for display with the custom HMD test pattern display software. Test pattern bit depth was set to 8 bits to represent the rendering pipeline of the ĒLVIS HMD software. Test patterns TG18-QC, TG18-CT, TG18-MP from the AAPM TG18 display evaluation report were used to provide measures of grey scale resolution and quality. A custom color brightness chart was used to measure perceived brightness steps in grayscale and 9 separate color hues, in 20 brightness steps of 5% each. A $16\times 12$ checkerboard pattern was displayed in the HMD at configurable angular offsets for geometric distortion and a corresponding scale copy was printed for object positioning evaluations.

### F. Subjects

#### 1) Bench Testing

A total of 10 engineering and non-engineering participants were selected for the evaluation of human in the loop performance criteria. All participants had at least some previous use of the Microsoft HoloLens HMD. 5 participants used corrective lenses (contacts or eyeglasses), 1 participant had Lasik corrective surgery, and 1 participant was red green colorblind. Participant ages ranged from 20 to over 65. All 10 participants completed the dynamic range and qualitative image evaluations, including one practicing electrophysiologist. 2 participants completed the object positioning evaluation; 1 with corrective lenses, and one with Lasik and both participants completed IPD calibration before conducting the evaluation.

#### 2) Observational Clinical Study

Patients who were scheduled to undergo an EP study and possible transcatheter ablation were identified from 7/2017–8/2017. Patients were contacted 2–3 weeks prior to their scheduled procedure and informed as to the study design. For those patients who expressed interest in participating in the study, informed consent (obtained from parents and participants > 18 years of age) and assent (from children ages 6–18 years of age) were obtained on the day of procedure. Inclusion criteria for the study were as follows: 1) pediatric patients between 6–21 years of age, 2) normal cardiac structure (as confirmed by 2D echocardiography), and 3) a macro-reentrant electrophysiologic substrate, such as atrioventricular reciprocating tachycardia (AVRT) including Wolff-Parkinson-White (WPW) and unidirectional concealed accessory pathways (AP), and atrioventricular nodal reentrant tachycardia (AVNRT). Patients were excluded for the following: 1) presence of major congenital heart disease, or 2) mechanical support.

Patient demographic data was collected prior to procedure as well as intraprocedural data. Metrics from the ĒLVIS were collected including battery discharge rate of the system, number of polygons displayed, frame rate of display and latency and is presented in **Table 1**.

**TABLE 1:** There Were 10 Patients Enrolled in the Observational Study With an Average Age of 13±4.5 Years, Weight of 55.5±27.8 kg, Height of 154.2±24 cm, and Body Surface Area of 1.5±0.5 m^2^ With 60% of Participants Being Female. Electrophysiology Substrates Included 3 Patients With Atrioventricular Nodal Reentrant Tachycardia (AVNRT) and 7 Patients With Accessory Pathway (AP) Mediated Tachycardias (6 Manifest, 1 Concealed). Of the 7 Patients With AP Mediated Tachycardias, There Were 6 Left Sided APs (1 Concealed, 5 Manifest) and 1 Right Sided AP (Manifest). All 10 Patients had Geometry Creation of the Right Atrium and Coronary Sinus. Six (60%) had Left Atrial Geometries Created in Addition to the Right Sided Structures. Local Activation Timing (LAT) Maps Were Created in 7 Patients (7 Patients With AVRT), and Voltage Maps Were Created in 5 Patients (3 Patients With AVNRT and 2 Additional Patients With AVRT). On Qualitative Assessment Between the LAT and Voltage Maps Generated in the EAMS as Compared to ĒLVIS, the Maps Were Equivalent With Regards to Differentiability of Color Texture. There Have Been No Acute or Chronic Adverse Events and No Clinical Recurrences of Tachycardia

Patient/Engineering Characteristics	ĒLVIS
Number of patients enrolled	10
Age (in years)	13±4.5
Weight (in kg)	55.5±27.8
Height (in cm)	154.2±24
Body Surface Area (in m^2^)	1.5±0.5
Electrophysiologic Diagnoses AVNRT Right sided accessory pathway mediated AVRT Left sided accessory pathway mediated AVRT	3 1 6
Geometries Created Right Atrium Coronary Sinus Left Atrium	10 10 6
Maps Created Local Activation Time Map (LAT) Voltage Map	7 5
Polygons	6K–12K
Frame Rate	30–60 frames/second
Battery Runtime	191 minutes
Latency	< 131 ms

### G. Statistical Analysis

Descriptive statistics are presented as mean averages with standard deviation in the Supplement. Statistical significance was set for p <0.05. All statistical calculations were performed in Matlab (R2017b).

## III. Results

### A. Geometric Distortion

The distribution of geometric errors across all images and poses was measured, resulting in a mean error of 0.52±0.31% SD, as illustrated in **Fig. 2b**. The distortion measured was below AAPM-TG18 guidelines of less than 5% for secondary class displays, and less than 2% guidance given for primary displays.

### B. Human-in-the-Loop Virtual Object Positioning

Two error measures were assessed: 1) differences in angulation, and 2) the error in distance from the perceived object, termed “scale”. The optical tracker measurements showed closely grouped positioning for each angulation of the object (**Fig. 2d**). The ratio between tracker measured distance and HMD stereoscopic rendered distance provided the measure of scale error. The scale error results showed a mean accuracy of 2.7%±1.2%, with a maximum of 6.2%, and a slight bias towards positive scale (farther away) for all users and devices. Although the AAPM-TG18 does not provide guidelines for perceived scale of virtual objects, the guidelines for geometric consistency can be used as a corollary. By further calibrating the constant bias, the perceived scale error is likely to be reduced below 5% to meet geometric distortion guidelines for secondary class displays and the 2% guidance given for primary displays may be achievable.

### C. Dynamic Range

There is a noticeable degradation in perceived dynamic range as the ambient lighting is increased. Overall, the average decrease in brightness level discrimination from dark lighting (3.5 cd/m^2^ average at the HMD) to normal ambient lighting (83 cd/m^2^ average at the HMD) for each color was 3.7 out of 20 brightness levels. It was observed that darker levels of blue hues were harder to differentiate than the darker levels of other color hues (average of 14.3 levels for blue vs an overall average of 18.0 out of 20 levels). There appeared to be no material difference in the ability to perceive brightness steps of particular hues for the lone colorblind test subject.

Grey level discrimination followed a similar reduction in perceived dynamic range with an average decrease in grey level perception of 2.6 out of 16 brightness levels from dark lighting to normal ambient lighting conditions. An average of 15.7 out of 16 grey levels were perceptible under darkened conditions, versus 13.1 levels under lighted conditions. The decrease in perception occurs in the darker levels of grey.

### D. Hardware Performance

*Latency:* The average additional latency from EAMS screen to ĒLVIS display was 68±42 ms. This latency includes EAMS delay in sending as well as processing time within ĒLVIS in translation, encoding, transmission, decryption, and display. The maximum latency observed was 147 ms. After analysis of packet captures, this anomaly was determined to be a wireless network retransmission recovery error between the EAMS and ĒLVIS. After corrective software modifications, further evaluation with a simulated EAMS did not reproduce this type of network error. This latency is near the acceptable range of 100 ms, based on human reaction time and the tolerance for latency in traditional human-computer interfaces [23]. Planned improvements to the networking infrastructure and to the test fixture itself can reduce the overall latency as well as the accuracy of these latency measurements.

#### 1) Frame Rate

For the tests performed, the average model complexity transmitted through the system was 12,899 polygons composed from 9,732 vertices. The aggregate mean frame rate was 53.3±5.4 frames/second. This frame rate meets the acceptable frame rate for EP display.

#### 2) Battery Runtime

Battery runtime on the HMD while replaying a simulated case, with no supplemental battery, demonstrated 234±17.6 minutes of runtime (**Figure 5d**). Attaching a supplemental battery of either 10,000mAh (2.4A at 5V, 38 Wh) or 13,000mAh (3A at 5V, 48.1 Wh) extended runtime duration to an average of 474.2 min or 483.2 min respectively. When connected to the original equipment manufacturer charger (2.5A at 5.2V), the device was able to charge while replaying the simulated case. This demonstrated that with the appropriate selection of charging devices, the duration could be extended using an off the shelf supplemental battery to 8 hours, or longer with an appropriately designed custom supplemental battery.

### E. E2 Study

The study generated data for evaluation of performance and usability from 10 total patients. (See Table 1) The electroanatomic map polygon count is a surrogate marker for the complexity of the cardiac geometry as it increases in size and detail over the course of the procedure as the map geometry is continually added to during catheter navigation. The resulting electroanatomic maps had a range in complexity from 6K-12K polygons and were rendered consistently at a rate of from 30–60 frames/second. Polygon count is the main factor influencing display rendering performance. Given the increasing complexity of mapping catheters, and the advent of high-density mapping, the capability to display increasingly higher polygon counts will remain of paramount priority.

To assess frame rate, selected geometries from four of the more complex case geometries were selected and played back through a simulation to represent high-load cases for the frame rate tests **(Fig. 5b)**. The maximum transmission latency measured from these playback recordings was 131 msec.

**FIGURE 6.**
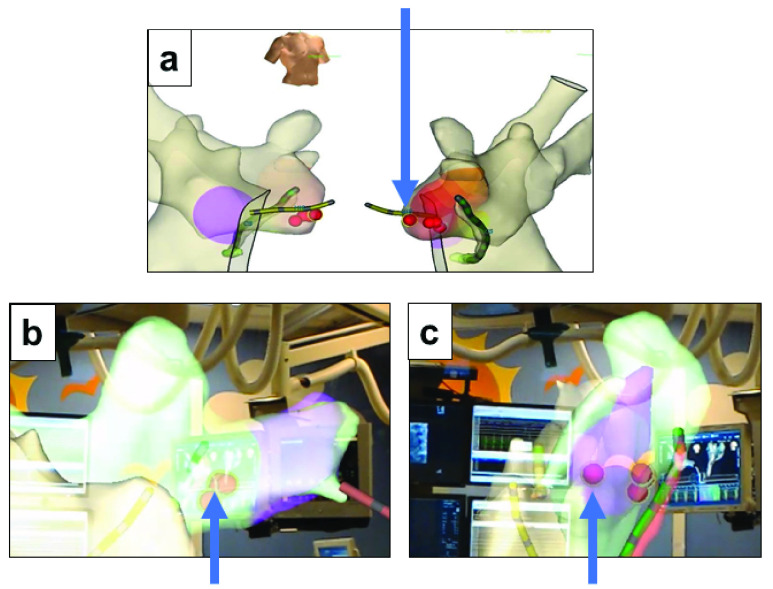
Perception of Anatomy: EAMS image (a) in right anterior oblique and left anterior oblique orientations as normally viewed during the procedure. Perception during procedure was that markers were well consolidated. View in ĒLVIS after performing physician visualized markers in stereoscopic 3D (b) and after rotation into a clearer view (c) revealing a greater distance between marker (indicated by arrow) within the marker cluster than previously interpreted.

The observing physicians demonstrated the ability to view various orientations of the anatomy as well as the catheter locations within the anatomy. In one case, the performing physician noticed a discrepancy in their original understanding of marker placement upon post-case review within the ĒLVIS. During review in the system, the physician immediately noticed that the marker placement was not as well clustered as originally understood and investigated further by rotating the data into a more direct view (**Fig. 6**). Each marker notes where an ablation lesion had been placed. The intent during the procedure had been to place additional “insurance” lesions on the site of success, but of the markers was clearly several mm from the rest of the marker cluster. This 3D relationship of the markers had not been well understood during the procedure and had the potential to impact patient outcome. Based on the point inaccuracy demonstrated in this patient, which was easier to interpret in ĒLVIS than on standard EAMS, we hypothesized that improved visualization may improve point accuracy, and will form an aim of future clinical studies.”

## IV. Conclusion

This the first report to describe a set of testing parameters to evaluate a mixed reality heads up display for medical use in a specific field, based on previously published medical imaging display parameters including human in the loop testing to evaluate near-eye 3D stereoscopic displays. Assessing human in the loop testing for a head up display is significant given that the 3D perception of depth may lead to decision making that may ultimately affect delivery of care in digital overlay applications. Some of the tested parameters, including accuracy and display distortion, will be applicable across extended reality applications. Other parameters will be medical sector specific, in this case to cardiac electrophysiology, such as latency and frame rate.

In 2019, AAPM-TG18 guidelines [22] were updated and released in the AAPM-TG270 guidelines [**?**] in response to advances in hardware display technology. These updates did not encompass the application of 3D displays or extended reality HMDs, and the use and evaluation of these novel technologies do not have standard tests or criteria. The tests described above form the basis of performance tests and metrics to evaluate mixed reality HMD technologies for intraprocedural use during minimally invasive procedures. It is important to note that although this testing does identify some challenges of current hardware in supporting image overlay, this capability is not a requirement of cardiac EPS procedures. Furthermore, the testing methods may be similar for the evaluation of HMDs in other medical applications, but the acceptability of the performance of the device is highly dependent on the user and the application, and should be validated through use.

The current testing described above demonstrates that the accuracy and performance characteristics of the Microsoft HoloLens HMD are able to support clinical use during cardiac ablation procedures. EAMS information could be displayed at a level at least equivalent qualitatively and quantitatively to an EAMS 2D display. Future studies will evaluate if the display can result in greater accuracy of catheter guidance and simplify procedures through reduced mental workload and interactions with EP lab personnel.

While the tests demonstrated adequate performance for cardiac ablation procedures, there are some notable limitations. The HoloLens HMD display provides an illusion of depth by providing stereoscopic imagery at a fixed focal plane. However, real physical objects in the same field of view are often at varying depths, resulting in perceived depth cue conflicts known as the vergence-accommodation conflict. In natural vision, the eyes perceive imagery that depends on both the distance of the perceived object from the observer, perceived by scale and focus cues (accommodation), and the distance between the images as perceived by the two eyes (vergence, or binocular parallax).

During human-in-the-loop placement of virtual objects, it was noted that despite individual IPD calibration, the “scale” or perceived depth of objects appeared to have an inherent bias. For the current application in cardiac EP, there are no current requirements for precise positioning of objects to correspond with real-world measurement dimensions. However, other medical applications that require superimposing reference graphics over a real object, may be much more sensitive to errors in depth perception. This in combination with vergence and accommodation issues may increase the occurrence of headache, nausea, discomfort, and/or reduced concentration. Careful attention to human factors designs and improved IPD calibration can mitigate this potential source of user discomfort and warrants further study for those applications.

Many medical imaging modalities (i.e. X-ray, MRI, CT, ultrasound, etc.) are displayed in grayscale. Current OST HMDs are not particularly well suited for viewing such monochrome images because the additive displays cannot render black, which is purely transparent on the HMD. Real-world objects are seen behind the image in the darker areas of the image and can affect interpretation. The use case of cardiac electroanatomic maps, however, are polychromatic in nature, both in the geometry as well as activation and voltage maps, and compatible with the current display capabilities of the HoloLens OST HMD.

The measured residual distortion error of 1.6% suggests that the visualization of the geometries will not be significantly altered by the ĒLVIS display and is acceptable based on current guidelines. No discrepancies were noted by the Observing Physician during the E2 study, whereas the benefits of depth perception for interpreting the positional catheter information relative to the cardiac geometry were noted. Each HMD display technology, including future iterations of existing platforms (e.g. HoloLens 2, Microsoft), presents unique compromises and challenges in brightness, color consistency, and resolution of the display, and may require reassessment for each specific medical application.

Although the necessary frame rate for geometrical models containing approximately 10,000 polygons was readily achievable with ĒLVIS, significantly more detailed and complex geometries may challenge the current ĒLVIS graphics processing pipeline and HMD hardware. Future development efforts would benefit from close collaboration with EAMS manufacturers to tailor these geometries for transmission and processing by HMD hardware to maintain frame rate and further reduce latency.

HMD hardware will continue to innovate at a rapid pace and as new processing, communication, and display technology is introduced, the evaluation of these systems will continue to become more complex. Although mobile phones have begun to establish a pathway for the use of consumer technology for medical applications, AR will continue to present unique challenges for evaluation. Despite these technical questions, preliminary clinician evaluation in the E2 study indicates that currently available off the shelf AR hardware may provide measurable benefits versus current displays in the interpretation of 3D data. By evaluating the performance metrics specific to the use case and the needs of the user, these methods and results presented provide a framework for adapting the existing display guidelines for the evaluation of these novel augmented reality systems to meet the specific requirements of each intraprocedural medical use and accelerate adoption.
